# Psychotic experiences in the general population, a review; definition, risk factors, outcomes and interventions

**DOI:** 10.1017/S0033291722002550

**Published:** 2022-11

**Authors:** Lorna Staines, Colm Healy, Helen Coughlan, Mary Clarke, Ian Kelleher, David Cotter, Mary Cannon

**Affiliations:** 1 Department of Psychiatry, Royal College of Surgeons in Ireland, Dublin 2, Ireland; 2 Department Psychology, Royal College of Surgeons in Ireland, Dublin 2, Ireland; 3 Department Psychiatry Beaumont Hospital, Dublin 9, Ireland; 4 School of Medicine, University College, Dublin 4, Ireland; 5 Lucena Clinic Child and Adolescent Mental Health Service, Rathgar, Dublin 6, Ireland

**Keywords:** Intervention, psychosis, psychotic experience outcomes, psychotic experience risk factors, psychotic experiences, psychotic-like experiences, youth mental health

## Abstract

Psychotic experiences (PE) are common in the general population, in particular in childhood, adolescence and young adulthood. PE have been shown to be associated with an increased risk for later psychotic disorders, mental disorders, and poorer functioning. Recent findings have highlighted the relevance of PE to many fields of healthcare, including treatment response in clinical services for anxiety & depression treatment, healthcare costs and service use. Despite PE relevance to many areas of mental health, and healthcare research, there remains a gap of information between PE researchers and experts in other fields. With this review, we aim to bridge this gap by providing a broad overview of the current state of PE research, and future directions. This narrative review aims to provide an broad overview of the literature on psychotic experiences, under the following headings: (1) Definition and Measurement of PE; (2) Risk Factors for PE; (3) PE and Health; (4) PE and Psychosocial Functioning; (5) Interventions for PE, (6) Future Directions.

## Objectives

Psychotic experiences (PE) are common phenomena in the general population (van Os & Linscott, 2012). PE are associated with a 4-fold increased risk of psychotic disorder & a 3-fold risk for mental disorders (Healy et al., 2019a). PE are also associated with increased healthcare costs & mental health service use (Bhavsar, McGuire, MacCabe, Oliver, & Fusar-Poli, 2018b; Rimvall et al., 2020). A recent study of a public mental health service for depression and anxiety found the presence of PE predicted higher psychopathology, and subsequent rate of recovery within the service, measured by number of sessions needed (Knight et al., 2020).

Greater awareness and inclusion of PE in healthcare research and clinical practise is an important step in expanding this field, and a general overview of PE research may be a valuable step to this goal. In this narrative review we aim to cover a broad range of subjects but, where possible, the information is synthesised from systematic reviews of the area (*k* = 28). Here PE are discussed under 6 headings; (1) Definition and Measurement of PE; (2) Risk Factors for PE; (3) PE and Health; (4) PE and Psychosocial Functioning; (5) Interventions for PE (6) Future Directions.

### Definition and measurement of psychotic experiences

#### Definition of PE

Within this review, PE is used to describe only hallucinations and delusions which occur at a sub-clinical level to those in the general population. This term is commonly used, however it creates categorical division of a continuum. The concept of the ‘extended psychosis-phenotype’, hypothesises that all psychotic phenomena exist on a continuum (van Os, 2003; van Os, Linscott, Myin-Germeys, Delespaul, & Krabbendam, 2009). At one end are individuals with psychotic disorders (severe and frequent hallucinations/delusions). At the other are individuals in the general population who experience occasional mild hallucinations/delusions. Evidence supports this fully dimensional scale of psychosis, showing evidence of shared environmental, genetic and neurobiological factors (DeRosse & Karlsgodt, 2015; Johns & Van Os, 2001; van Os et al., 2009).

Despite this robust evidence, research on subclinical psychotic phenomena commonly use categorical definitions e.g. PE or those at clinical high risk for psychosis (CHR). Both are forms of subclinical psychotic symptoms, and share common symptomology (Fusar-Poli et al., 2013; Kelleher & Cannon, 2011). Proposed delineations exist, such as those at CHR showing help-seeking behaviour (van Os & Linscott, 2012). However, CHR have been found in non-help-seeking populations (Staines et al., 2021; Uhlhaas et al., 2017), and those reporting PE show help-seeking behaviour (Armando et al., 2012), making this differentiation insufficient. Similarly, distress about symptoms as a delineation (Johns et al., 2014), is vulnerable to individual differences (Coughlan et al., 2020).

Additionally, PE as defined within this review does not discuss subclinical negative symptoms, cognitive deficits, thought disorder or affective disorder as PE. This is as in the literature, they are not commonly examined as ‘PE’. When both are included they are often examined as a wider phenomenon e.g. ‘psychotic experiences and negative symptoms (PENS)’ in (Pain et al., 2018).

#### Occurrence of psychotic experiences in the general population

The projected lifetime risk of PE is 7.8% (McGrath et al., 2016). However, prevalence differs across a lifetime, at around 5–7% in adulthood (Linscott & van Os, 2013; van Os et al., 2009; Yates et al., 2021), but a significantly higher prevalence in children (17%) and moderately higher in adolescence (8%) (Kelleher et al., 2012). A study of PE across a lifetime observed this declining trend, finding the lowest prevalence in later adulthood (⩾ 70 years) of 3% (Yates et al., 2021). However (Linscott & van Os, 2013) examined methodological variables and observed reporting type (self-report/clinical interview) accounted for 19.7% of the variance in prevalence rates of PE. Similarly, different types of PE may have differing prevalence rates, e.g. a systematic review of auditory PE found there were equal prevalence in children (12.7%) and adolescence (12.4%) (Maijer, Begemann, Palmen, Leucht, & Sommer, 2018).

Less is known about incidence, one review yielded estimates between 2.5% and 3% median incidence rate across a few (*k* = 6) studies (Linscott & van Os, 2013; van Os et al., 2009). Patterns for difference in age are consistent in adulthood; one large-scale adult sample showed a decline in incidence from early to late adulthood (Tien, 1991). Intriguingly, one study in a child and adolescent sample found the incidence was higher in adolescence than early childhood (Sullivan et al., 2020). A systematic review of hallucinatory PE found a similar result of incidence rate per-person year range of 0.1–1.3% in children and adolescents (Rubio, Sanjuán, Flórez-Salamanca, & Cuesta, 2012). Reasons for differences between prevalence and incidence are currently unknown, although methodological differences must be considered (Linscott & van Os, 2013; Maijer et al., 2019a).

For most individuals (~80%) PE are transient, (Linscott & van Os, 2013; McGrath et al., 2015). For the remaining (~20%) PE may recur. Individuals who experience multi-modal PE are more likely to experience recurring PE (McGrath et al., 2015). Studies commonly describe PE which occur repeatedly as ‘persistent’ (e.g. van Os et al., 2009). However, even in those with repeated incidence of PE, these symptoms remit for significant lengths of time (McGrath et al., 2015). Therefore this paper has opted for the term ‘recurring PE’ which we believe better reflects the phenomena.

#### Challenges in researching psychotic experiences

Two key challenges of PE research: phenomenology & measurement.

Hallucinations and delusions vary widely in phenomenology, e.g. auditory hallucination and paranoid ideation are both common PE (Armando et al., 2010; Coughlan et al., 2020). Additionally, subtypes of PE may represent different trajectories, with evidence finding bizarre experiences and perceptual abnormalities were associated with higher distress, depression and worse functioning (Armando et al., 2010). Some PE subtypes are more associated with suicidal behaviour (Hielscher et al., 2020, 2021).

There is significant heterogeneity in measurement tools; (Lee et al., 2016) reported that across 76 studies, 41 different assessment tools were used. There is some suggestion that self-report measures may generate more false-positive ratings of PE (Schultze-Lutter et al., 2014; van Os et al., 2009), and have been found to report higher rates of PE than clinical interview (Healy et al., 2019a). One study comparing the Development-and-Well-Being Assessment (DAWBA) self-report tool to a clinical interview observed that the sensitivity (73.8%), specificity (77.1%), and the negative predictive value (96.3%) were good, but positive predictive value (26.8%) was low (Gundersen et al., 2019). Additionally, sensitivity (10.0–43.1) of symptom subtype was poor for the DAWBA (Gundersen et al., 2019). However, self-report and clinically validated PE show similar outcomes (Healy et al., 2019a), and ‘false positive ratings’ of PE were still found to be associated with psychiatric outcomes (van der Steen et al., 2019).

Additionally, self-report measures without information on duration, frequency, or conviction risk misidentifying events. One large-scale sample found that of those identified with self-report PE, 11% also met criteria for CHR, and a subsample (*n* = 15) met criteria for overt psychosis (Moriyama et al., 2019). Questions on auditory hallucinations appear to be the most valid single-item measure of PE within child and adolescent populations (Kelleher & Cannon, 2011). Although (Leiderman, 2011) found visual hallucinations were more frequent in their sample, and the DAWBA found visual hallucinations had a higher predictive value (Gundersen et al., 2019). Overreliance on single-items has limitations; it restricts research to a symptom subtype, and a single item endorsement is likely to produce false negatives.

### Risk factors of psychotic experiences

#### Heritability and genetics

Sex differences have been examined to mixed results, one systematic review did observe higher rates in males (van Os et al., 2009), although a second review and a large international sample both failed to replicate this (Linscott & van Os, 2013; McGrath et al., 2015). One systematic review for heritability & genetics and PE exists, but was limited by a small number of studies (*k* = 13) (Ronald & Pain, 2018). Single-nucleotide polymorphism (SNP)-studies failed to find evidence of heritability for PE (Ronald & Pain, 2018). Genome-wide association studies (GWAS) (Ortega-Alonso et al., 2017; Pain et al., 2018) found evidence of genome-wide significant association, but neither was able to independently replicate these findings (Ronald & Pain, 2018). Newer studies on GWAS have expanded these findings showing genetic overlap between PE and psychiatric disorders including schizophrenia and bipolar disorder (Barkhuizen, Pain, Dudbridge, & Ronald, 2020; Legge et al., 2019). Poly-genetic risk scores were the most studied in the systematic review (*k* = 10) (Ronald & Pain, 2018) which showed moderate evidence for overlapping genetic risk for schizophrenia and PE. Investigations into gene-environment interactions indicate some SNP and risk haplotypes interact with environmental factors of bullying (Cristóbal-Narváez et al., 2016), drug use (Zammit, Owen, Evans, Heron, & Lewis, 2011), and childhood trauma (Ramsay et al., 2013).

#### Pre/perinatal complications

Higher birth weight has been associated with a reduced risk of PE (Thomas et al., 2009), and lower birthweight with increased risk (Drakesmith et al., 2016). Maternal infections, negative life events, cannabis use and smoking during pregnancy have been identified as a risk factor for PE (Dorrington et al., 2014; Fine et al., 2019; Zammit et al., 2009). Maternal binge drinking has not been linked with PE (Gregersen, Dreier, & Strandberg-Larsen, 2020), but large weekly consumption of alcohol was (Zammit et al., 2009). Both studies used large samples and collected drinking data during pregnancy, allowing for solid comparability. Older paternal age is associated with risk for PE (Foutz & Mezuk, 2014). Winter season of birth shows a moderate risk for PE in meta-analysis (Córdova-Palomera et al., 2015).

#### Early development and health factors

Children exposed to Epstein-Barr Virus or atopic conditions in childhood increase risk of adolescent PE (Khandaker, Pearson, Zammit, Lewis, & Jones, 2014). Similarly, childhood dysregulation of inflammatory markers (interleukin 6 and C-reactive protein) and certain complement proteins (C1RL, C5, CFH) have been identified as an early biomarker for later development of PE (Föcking et al., 2019; Khandaker et al., 2014; Mongan et al., 2021). These results suggest a role for the early-life and immune system development in vulnerability to PE.

#### Cognitive dysfunction

Ross, McKay, Coltheart, and Langdon (2015) in a systematic review found that individuals with subclinical delusions reported worse data gathering abilities in ‘jumping to conclusions’ (JTC) measures. A much broader systematic review and meta-analysis conducted by Livet, Navarri, Potvin, and Conrod (2020) found PE in the general population were strongly associated with aberrant salience, moderate trends in external attribution biases and attention to threat, and weak evidence for JTC and belief inflexibility. However heterogeneity between studies was significant (Livet et al., 2020). Other studies have observed additional cognitive dysfunction such as motor speed, and IQ (Carey et al., 2019; Horwood et al., 2008), and evidence suggests these can be observed from early infancy (Carey et al., 2021). However given heterogeneity between studies further analysis is needed, and a greater understanding of why these results differ between groups.

#### Neuroanatomical development

A range of subtle neuro-anatomical differences have been observed in those reporting PE. These include, global and localised differences in grey matter volume and white matter integrity (Dooley et al., 2020; Satterthwaite et al., 2015), and widespread structural and functional differences across the frontal, temporal, & parietal lobes, and basal ganglia, all regions strongly implicated in psychosis (Drakesmith et al., 2016; Jacobson et al., 2010; Modinos, Ormel, & Aleman, 2010; Okada et al., 2018). Additionally, widespread functional dysconnectivity (default mode dysfunction in particular, but with increasing interest in the motor network) and network efficiency deficits (Drakesmith et al., 2015; Jacobson McEwen et al., 2014; Karcher, O'Brien, Kandala, & Barch, 2019; Orr, Turner, & Mittal, 2014; O'Neill et al., 2020). However, it is currently unclear whether the differences in brain structure and functioning precede or follow the onset of PE.

#### Urbanicity

Individuals living in densely populated areas have been shown to have a higher risk of PE (Linscott & van Os, 2013; van Os et al., 2009). Research on air pollution found it was a significant modifier and accounted for 60% of the variance (Newbury et al., 2019). Additionally, urbanicity and PE in low and middle-income countries failed to show an association (DeVylder et al., 2018b). One large-scale study in China found rural upbringing was associated with elevated risk for PE (Wang et al., 2019). This study identified social stress and economic deprivation was higher in rural China, both of which show increased risk of PE (DeVylder et al., 2016). Negative perception of neighbourhood has also been linked to PE (Newbury et al., 2017). These findings suggest culturally dependent differences in urban/rural life, pollution and perception, may underpin the mechanisms by which urbanicity can elevate risk of PE.

#### Ethnic minority status

Research on ethnic minority status (EMS) & migrant status and PE indicate that there are differences based on EMS, with high discrepancies between groups. One meta-analysis found certain EMS groups showed higher PE in certain regions (Leaune et al., 2019), even when sociodemographic variables were accounted for. However the high heterogeneity between different EMS groups suggested discrimination and deprivation of certain EMS groups might better explain this finding (Leaune et al., 2019). A second meta-analysis supported this by showing that accounting for psychological stress and clinical diagnosis, in addition to sociodemographic variables, attenuated these findings (Tortelli et al., 2018).

A systematic review of discrimination observed experiencing discrimination increased risk for PE, particularly paranoia (Pearce, Rafiq, Simpson, & Varese, 2019). A second US-based review observed negative police interactions increased risk for PE and other mental disorders in black Americans (McLeod, Heller, Manze, & Echeverria, 2020), although only one study specifically examined PE (DeVylder et al., 2018a). Both (Leaune et al., 2019; Tortelli et al., 2018) failed to find evidence that migrant status alone indicated increased risk of PE. One meta-analysis found residential concentration of own ethnic identity was shown to be a protective factor against PE among EMS (Bécares, Dewey, & Das-Munshi, 2018).

#### Socio-economic status

Lower socio-economic status (SES) is associated with higher rates of PE (Loch et al., 2017; Mamah, Mutiso, & Ndetei, 2021; van Os et al., 2009). As with urbanicity, research has begun to examine the mechanisms which might produce these differences; One study found social disadvantage, discrimination, and unstable housing situations increased SES predictive value (Veling & Adriaanse, 2013). Another examining PE in children found neighbourhood deprivation and poverty were both significantly associated with PE (Karcher, Schiffman, & Barch, 2021b).

#### Trauma

Trauma such as sexual, physical, or emotional abuse increases risk of PE (Daalman et al., 2012; Fisher et al., 2013). Studies have demonstrated a dose-response rate between traumatic experiences and PE (Coughlan & Cannon, 2017; Shevlin et al., 2011). A recent meta-analysis observed distinctive pathways between trauma and subsequent PE subtype, driven by differing psychological processes (e.g. post-traumatic symptomology, attachment and social cognition) (Bloomfield et al., 2021). However this analysis was limited by the significant number (*k* = 14 of 22) of cross-sectional designs of included studies.

There is substantial evidence of a link between bullying and PE (Fisher et al., 2013; Wolke, Lereya, Fisher, Lewis, & Zammit, 2014). Within a longitudinal study of adolescents, Kelleher et al. (2013) observed a bidirectional relationship between bullying and rates of PE. Evidence indicates multi-victimisation results in higher rates of PE (Arseneault et al., 2011). Research into paranoid delusions has shown direct causal links to negative affect, bullying, peer difficulties, and negative behaviours on social media (Bird, Evans, Waite, Loe, & Freeman, 2019).

#### Psychopathology

A large systematic review of 15 studies found a majority showed internalising and externalising behaviours in childhood and adolescence increase risk of PE (Gin, Stewart, & Jolley, 2021). Research using a large longitudinal sample of children and adolescents has demonstrated a bi-directional relationship between psychopathology and PE (Healy, Coughlan, Clarke, Kelleher, & Cannon, 2020). Insomnia and excessive daytime somnolence have been linked to higher rates of PE (Barton, Kyle, Varese, Jones, & Haddock, 2018; Reeve, Sheaves, & Freeman, 2015). Evidence suggests a bi-directional relationship between insomnia and PE (Reeve, Nickless, Sheaves, & Freeman, 2018).

#### Smoking tobacco or cannabis

Tobacco use has been shown to increase risk for PE (Bhavsar et al., 2018a). Shakoor et al. (2015) found use of cannabis and the presence of PE had shared environmental risk factors. A systematic review found younger age, and frequent cannabis use are associated with higher reports of PE (Ragazzi, Shuhama, Menezes, & Del-Ben, 2018). Maternal cannabis use during late-term pregnancy increased psychosis-proneness (Fine et al., 2019). Kuepper et al. (2011) demonstrated continued cannabis use was associated with a greater risk of recurring PE.

#### Risk factors for recurring psychotic experiences

Although evidence supports that recurring PE are associated with poorer outcomes (Bhavsar et al., 2021; Rimvall et al., 2021), currently less is known on recurring risk factors. Evidence has suggested that recurring PE are not associated with family history of psychosis (Karcher et al., 2021a; Thapar et al., 2012), although one study did observe a moderate trend (Karcher et al., 2021a). Studies examining sex have found more women report recurring PE; using latent growth modelling techniques higher numbers of female participants were in their ‘persisting’ & ‘increasing’ PE groups (Mackie, Castellanos-Ryan, & Conrod, 2011; Thapar et al., 2012), and other forms of analysis have found a similar gender disparity (DeVylder, Lehmann, & Chen, 2015; Fonville et al., 2019). Early developmental problems including birth complications, maternal smoking and infections were associated with higher rates of recurring PE (Rössler et al., 2007; Thapar et al., 2012). Cognitive deficits are underexplored in the literature, but currently show no association with recurring PE (Kalman, Bresnahan, Schulze, & Susser, 2019).

EMS was associated with recurring PE, one study in an American sample (Calkins et al., 2017) found higher rates of recurring PE in African American participants (76.6% *v.* 42.5% in controls). However a second study failed to find these differences (DeVylder et al., 2015). Other environmental factors including urbanicity and SES were not found to be related (Bourque, Afzali, O'Leary-Barrett, & Conrod, 2017; Peters et al., 2016; Wigman et al., 2011).

Behavioural measures such as frequent cannabis use, stressful-life events, trauma, victimisation and bullying have been associated with recurring PE (Bourque et al., 2017; Mackie et al., 2013; Sun et al., 2017; Wigman et al., 2011). Cougnard et al. (2007) further reported that recurring PE were associated with a higher number of cumulative environmental risk factors. Markers of mental ill health including more severe symptomology, higher medication prescriptions, insomnia, higher reported rates of anxiety and depression, and low functioning have all been linked to recurring PE (Calkins et al., 2017; Reeve et al., 2015; Yamasaki et al., 2018).

Despite a number of known risk factors, in a recent scoping review, Kalman et al. (2019) failed to find any consistent evidence of risk factors for recurring PE. Modelling techniques to develop predictive models of recurring *v.* transient PE found a similar limitation using current known risk factors (AUC = 0.66, 7.4% of variance, Sensitivity = 68.8%; Specificity = 54.4%) (Steenkamp et al., 2021), although the model did differentiate recurring PE from control.

### Psychotic experiences & health

#### Psychotic disorders

A systematic review of PE found adolescent PE had a 4-fold increased risk for developing psychotic disorder (Healy et al., 2019a), and this effect remained even after controlling for childhood psychopathology (Fisher et al., 2013). Recurring PE examined in a longitudinal study design (8.4 years) found recurring PE had increased risk of psychotic disorders relative to transient PE (Dominguez, Wichers, Lieb, Wittchen, & van Os, 2011).

#### Non-psychotic disorders

A meta-analysis of longitudinal prospective studies robustly demonstrated the relationship between non-psychotic disorders and PE (Kaymaz et al., 2012). Healy *et al*. (2019a, 2019b) found childhood PE was associated with a 3-fold increased risk for mental disorder. Conversely, Varghese et al. (2011) found the presence of major depressive or anxiety disorder was associated with 4-fold and 3-fold elevated risk for subsequent PE. One systematic review found PE subtype showed different associations, observing paranoia PE, but not grandiosity, was highly associated with anxiety (Bird, Waite, & Freeman, 2018). These associations are further elevated for those with recurring PE (Chan et al., 2021; Downs, Cullen, Barragan, & Laurens, 2013). Co-occurring PE and mental disorders is well documented (Downs et al., 2013; Kelleher et al., 2012; Laurens, Downs, Cullen, Barragan, & To, 2012). Evidence shows a majority of adolescents with PE meet criteria for at least one lifetime non-psychotic mental disorder, and had particularly elevated risk of psychiatric multi-morbidity (Armando et al., 2010; Laurens et al., 2012). The presence of PE in those with mental disorders were associated with more severe symptoms of anxiety and depression, and showed slower recovery rates (Knight et al., 2020).

Psychotic experiences are associated with subsequent psychopathology, with the presence of PE being associated with higher rates of substance disorders, internalising & externalising symptoms in adolescence (Cederlöf et al., 2017), and early adulthood (Carey et al., 2021). PE are associated with escalating trajectory of psychopathology (Iorfino et al., 2019). Recurring PE is associated with worse psychopathology (Downs et al., 2013).

#### Healthcare needs

Research suggests PE are associated with an elevated risk for future psychiatric diagnosis, mental health service use, pharmacological treatment, healthcare costs and health-related quality of life (Rimvall et al., 2020, 2021). These findings were shown using the Copenhagen Child Cohort 2000, a longitudinal study design that at baseline represented 1 in 10 children born in Denmark (Olsen et al., 2020). This study had the advantage of access to The Danish National Health Service Register, allowing for comprehensive analysis of healthcare costs and use. In particular, the presence of PE and a co-occurring mental disorder were associated with a greater risk for these measures (Rimvall et al., 2020). Similar results have been found in other independent samples (Bhavsar et al., 2021), with one (Karcher et al., 2021a) observing that persistence and distress related to PE both increased mental health service use. A moderately sized (*k* = 13) meta-analysis observed individuals with PE showed a 2-fold increase for mental health service use compared to healthy controls (Bhavsar et al., 2018b). Additionally, studies have observed the presence of PE is associated with more medical conditions (e.g. asthma, arthris, hearing/visual problems) (Moreno et al., 2013). Most concerning, a longitudinal study in a large sample (*n* = 15 049) found that the presence of PE was associated with a 5 year median shorter lifespan (Sharifi et al., 2015), with neoplasms and death by suicide being significantly higher than those without PE at baseline.

#### Suicidal behaviour

Psychotic experiences are strongly associated with suicidal ideation, attempts and death by suicide (Yates et al., 2019). Systematic reviews and meta-analyses have indicated that those with PE were at a 2–3 fold increased risk of suicidal behaviours (Honings, Drukker, Groen, & van Os, 2016a; Yates et al., 2019). Recurring PE are associated with an even greater risk of suicide behaviour relative to transient-PE (Connell et al., 2016). One study of adolescents found depression mediated the relationship between PE and suicide (Nunez et al., 2020), and a review expanded on this observing significant mediating roles of mental disorders, psychological distress and environmental factors (Hielscher et al., 2021).

#### Mediators of psychotic experiences

To date substantially less is known on the mediators of PE. Research has identified a number of potential mediators between PE and risk/outcome factors. These mediators include parent-child conflict, self-concept, internalising problems, coping strategies and social and community cohesion (Crush, Arseneault, Jaffee, Danese, & Fisher, 2018; Dhondt, Staines, Healy, & Cannon, 2022; Healy et al., 2019b, 2020, 2021; McMahon et al., 2020).

### Psychotic experiences & psychosocial functioning

Evidence shows PE are associated with poorer global functioning (Carey et al., 2021; Healy et al., 2018; Kelleher et al., 2015). However, Brandizzi et al. (2014) observed only perceptual abnormalities were associated with worse functioning. Occupational functioning is poorer, with higher rates of unemployment (Scott, Chant, Andrews, & McGrath, 2006; van Os et al., 2009) and arrest (Honings et al., 2016a). Similarly worse social functioning has been associated with PE, including less social support (Saha, Scott, Varghese, & McGrath, 2012), greater externalising locus of control (Sullivan, Thompson, Kounali, Lewis, & Zammit, 2017), higher rates of divorce (Linscott & van Os, 2013), & perceived social stigma (Lien et al., 2015). Two large cross-sectional studies have observed higher rates of interpersonal violence (Kinoshita et al., 2011; Mojtabai, 2006), with one observing a dose response with higher numbers of PE increasing rates (Mojtabai, 2006). Subsequent longitudinal studies have observed an association between PE (self-report & clinically validated) and subsequent violence, but found adjusting for psychopathology and sociodemographic differences (trauma, negative life events, social support) substantially mediated the effect (Honings et al., 2016b).

PE during childhood and adolescence are associated with lower optimism, self-esteem, avoidant coping and school misconduct (Dolphin, Dooley, & Fitzgerald, 2015), poorer functioning (Calkins et al., 2017; Kelleher et al., 2015), and worse language &mathematical ability (Steenkamp et al., 2021b). PE during childhood have not been shown to have a sustained negative effect on education or interpersonal skills to adulthood (Coughlan et al., 2021; Rimvall et al., 2021).

### Interventions for psychotic experiences

Individuals who report PE and a co-occurring mental disorder show worse outcomes and poorer recovery (Bhavsar et al., 2021; Knight et al., 2020). Intervening early may reduce ‘risk’ in individuals who are known to be vulnerable. A key recent finding (Knight et al., 2020) found the presence of PE predicted slower recovery rates. This suggests PE might be a useful marker for determining the duration required for a cognitive intervention to be effective.

However, to date there have been very few interventions focused primarily on PE. Soneson et al. (2020) were unable to complete a meta-analysis on interventions for PE due to this deficit. Freeman et al. (2017) carried out a trial of digital cognitive behavioural therapy (CBT) for insomnia on a large sample (*n* = 1891), finding the intervention led to a reduction in PE. Two other samples, with significantly smaller samples, found traditional in-person CBT reduced PE frequency and impact (Maddox et al., 2013; Maijer, Staring, Bartels-Velthuis, Palmen, & Sommer, 2019b). One study using mindfulness did not find a reduction in PE (Langer, Cangas, & Gallego, 2010). This field is expanding, with new therapy approaches currently in progress e.g. (Ashford et al., 2022; Jolley et al., 2017).

### Future direction of research

Future direction for measurement: More focus should be placed on the different types of PE, to elucidate their potentially divergent trajectories and psychopathologic significance. This will require more extensive clinical characterisation of PE. Development of such tools are growing, the SOCRATES assessment of perceptual abnormalities and unusual thought content by (Kelleher & Cannon, 2016) is a semi-structured interview approach on the source, onset, duration, frequency, content, attributions, reality testing, timings, severity of distress and effects on functioning of PE. Other tools such as the Auditory Vocal Hallucination Rating Scale examine auditory PE by measuring content, duration, experience, intensity, anxiety, distress and changes in behaviour and function (Bartels-Velthuis, van de Willige, Jenner, & Wiersma, 2012).

Secondly, the criteria used to define PE should have an evidence based in clinical outcomes. One key way this can be clarified is by studies which consider the psychosis continuum, measuring not only within distinct categories but cross-spectrum differences (DeRosse & Karlsgodt, 2015). This would help address the limitations imposed by categories, and help unify current understandings of psychotic phenomena.

Future direction for analysis. A significant number of risk factors, and a modest number of protective and mediating factors, have been identified to date. However, knowledge of the mechanisms which underlie these events is unknown. It is likely that a number of factors interact on multiple-levels. It is possible that more advanced statistical techniques, such as network analysis or machine learning algorithms, may mimic this type of interactive environment better than the current techniques.

PE has a significant association with psychopathology (Gin et al., 2021). Some psychometric research (Stochl et al., 2015) has argued that symptoms of anxiety, depression and PE should be considered as a single dimension, with PE representing the more severe end of the spectrum (Van Os & Guloksuz, 2017). Longitudional studies have observed overlapping patterns of symptom changes in PE and anxiety/depression symptoms, which may support this model (Wu et al., 2021). Alternatively, PE and psychopathology could be viewed as part of a dynamic network of symptoms, which can develop into mental illness (Booij et al., 2018; Guloksuz et al., 2020; Nelson, McGorry, Wichers, Wigman, & Hartmann, 2017). New studies aimed at examining this interconnected relationship currently exist, e.g. the MIRORR study (Booij et al., 2018). Both approaches incorporate the high correlation between psychopathology and PE, and might more accurately reflect the complex system of developing mental ill health. Expanding our current conceptions of PE to include psychopathology, as well as other subclinical symptoms (negative symptoms, cognitive & affective deficits, thought disorder), may be a key step.

Future directions for PE treatment: While PE does not predict poor outcomes, it has the potential to be utilised as an early marker for mental disorders. This could be valuable as an early screening tool to identify vulnerable individuals outside of clinical services, as demonstrated by (Knight et al., 2020). Similarly, (DeVylder, Waldman, Hielscher, Scott, & Oh, 2020) found PE could be used to predict suicidal ideation. These findings and evidence of health care outcomes in those reporting PE (Bhavsar et al., 2021; Rimvall et al., 2020, 2021), all suggest PE may have a valuable role in early intervention. Interventions which include PE should be a growing area of priority, this could include; (1) PE as a measure of improvement following intervention, (2) reducing recurring PE as a primary aim of intervention, (3) Reducing rates of PE as a method to reduce severity of mental disorders, (4) Reducing PE as a long-term method to reduce healthcare needs and costs.

## Conclusions

The study of PE is a rapidly growing area of research, which has faced some significant difficulties in unifying definition, assessment and research. We hope this review has offered some clarity to the area, and provided a substantive overview on PE, their risk factors and outcomes, with the hope that future research may be a more unified body that can continue to push forward in this promising field.
